# Plasma Somatostatin Levels Are Lower in Patients with Coronary Stenosis and Significantly Increase after Stent Implantation

**DOI:** 10.3390/jcm13164727

**Published:** 2024-08-12

**Authors:** Balázs Sütő, József Kun, Teréz Bagoly, Timea Németh, Erika Pintér, Dorottya Kardos, Zsuzsanna Helyes

**Affiliations:** 1 Department of Anaesthesia and Intensive Therapy, Medical School, University of Pécs, 7624 Pécs, Hungary; suto.balazs@pte.hu; 2 Department of Pharmacology and Pharmacotherapy, Medical School, University of Pécs, 7624 Pécs, Hungary; 3 Hungarian Centre for Genomics and Bioinformatics, Szentágothai Research Centre, University of Pécs, 7624 Pécs, Hungary; 4 National Laboratory for Drug Research and Development, 1117 Budapest, Hungary; 5 Department of Languages for Biomedical Purposes and Communication, Medical School, University of Pécs, 7624 Pécs, Hungary; 6 Hungarian Research Network (HUN-REN-PTE), Chronic Pain Research Group, University of Pécs, 7624 Pécs, Hungary; 7 Department of Anaesthesia and Intensive Therapy, General District Hospital Szekszárd, 7100 Szekszárd, Hungary

**Keywords:** neuropeptides, inflammation, tissue damage, reperfusion, ischemic heart disease, stable angina, coronarography, stent implantation, enzyme-linked immunosorbent assay

## Abstract

**Background/Objectives**: Stimulated capsaicin-sensitive peptidergic sensory nerves release somatostatin (SST), which has systemic anti-inflammatory and analgesic effects, correlating with the severity of tissue injury. Previous studies suggest that SST release into the systemic circulation is likely to serve as a protective mechanism during thoracic and orthopedic surgeries, scoliosis operations, and septic conditions, all involving significant tissue damage, pain, and inflammation. In a severe systemic inflammation rat model, SST released from sensory nerves into the bloodstream enhanced innate defense, reducing mortality. Inflammation is the key pathophysiological process responsible for the formation, progression, instability, and healing of atherosclerotic plaques. **Methods**: We measured SST-like immunoreactivity (SST-LI) in the plasma of healthy volunteers in different age groups and also that of stable angina patients with coronary heart disease (CHD) using ELISA and tracked changes during invasive coronary interventions (coronarography) with and without stent implantation. Samples were collected at (1) pre-intervention, (2) after coronarography, (3) 2 h after coronarography initiation and coronary stent placement, and (4) the next morning. **Results**: There was a strong negative correlation between SST-LI concentrations and age; the plasma SST-LI of older healthy volunteers (47–73 years) was significantly lower than in young ones (24–27 years). Baseline SST-LI in CHD patients who needed stents was significantly reduced compared to those not requiring stents. Plasma SST-LI significantly increased two hours post stent insertion and the next morning compared to pre-intervention levels. **Conclusions**: Age-related SST decrease might be a consequence of lower gene expression within specific hypo-thalamic nuclei as has been previously demonstrated in rodent animals. Reperfusion of ischemic myocardium post-stent implantation may trigger SST release, potentially offering protective benefits in coronary heart disease. Investigating this SST-mediated mechanism could offer valuable insights for future therapies.

## 1. Introduction

Complex interactions of the nervous, endocrine, and immune systems collectively govern numerous physiological and pathophysiological processes including tissue injury and repair, inflammation, and pain [1]. Key regulators in these mechanisms include neuropeptides, hormones, lipid mediators, and cytokines. Neuropeptides released from the peripheral terminals of sensory neurons in response to various stimuli during tissue damage exhibit multiple actions on vascular endothelial and smooth muscle cells, inflammatory and immune cells, and neurons [2,3,4].

In the peripheral nervous system, somatostatin (SST) is present in sensory and sympathetic neurons and inflammatory cells and exerts inhibitory effects on nociception and inflammation [5,6,7,8,9]. SST-producing cells are predominantly neurons, immune cells, and endocrine-like cells in various organs [10,11]. SST regulates diverse physiological processes in the nervous and neuroendocrine systems; it modulates inflammatory cells such as lymphocytes, monocytes, macrophages, peripheral blood mononuclear cells, and thymocytes. The effects of SST go beyond its local impact, it reaches distant sites through the systemic circulation and exerts a broad range of inhibitory actions [6,7] via its five Gi-protein-coupled receptors (SST_1_–SST_5_) [10,11,12,13,14]. These receptors have been identified with high expression in neoangiogenic and peritumoral vessels, epithelial cells, proliferating synovial vessels, and activated lymphocytes and monocytes involved in inflammatory responses [10,12,13,14,15,16]. The activation of SST_1_ and SST_4_ is suggested to be involved in the anti-inflammatory and analgesic effects of SST [17]. We earlier published that plasma SST-like immunoreactivity (SST-LI) increases during and after abdominal [18] and thoracic and orthopedic surgeries [19]. Additionally, we also observed that plasma SST-LI levels increase during sepsis [20]. Throughout the entire operative procedure of scoliosis surgery, there was a significant increase in plasma SST-LI, which is likely to be due to extensive tissue damage. In contrast, no such increase was noted in disc hernia operations with minimal invasive intervention [21]. 

SST regulates multiple physiological functions in the cardiovascular system, such as modulating vascular tone and cardiac contractility in ischemic heart diseases, cardiac remodeling, and recovery after infarction [22]. Anatomical and functional findings demonstrate that capsaicin-sensitive peptidergic sensory fibers originating from both spinal and vagal pathways innervate the heart [23,24,25] and play a crucial role in the adaptation mechanisms to ischemic injury [26]. It has been stated that SST released from capsaicin-sensitive nerve terminals prevented retinal ischemia/reperfusion injury in a mouse model [27]. Recent research shows that SST exerts protective actions on cardiomyocytes during ischemia/reperfusion injury. Despite the presence of the SST peptide in the heart, mRNA was not detectable, which suggests its sensory neural origin [15]. SST released from sensory nerves may contribute to cardioprotection [28] mainly via SST_1_ and SST_2_, but other mechanisms cannot be ruled out [15].

In this study, we measured SST-like immunoreactivity (SST-LI) in the plasma of ischemic heart disease patients (IHD) with stable angina and determined its alterations during intervention procedures for coronary stent implantation in comparison with coronarography without stent insertion and healthy volunteers in different age groups. We aimed to establish a link between the severity of ischemia, the extent of myocardial damage, tissue reperfusion, and plasma SST levels.

## 2. Materials and Methods

### 2.1. Patients

Our prospective, descriptive research study involved 24 patients with IHD and stable angina undergoing elective coronary angiography. They had various diagnoses such as hypertension, previous myocardial infarction (MI), high blood lipid and cholesterol levels, and type II diabetes. These patients were prescribed a variety of medications, including antiplatelets, antihypertensives (beta-blockers, ACE inhibitors, and angiotensin II receptor blockers), diuretics, potassium supplements, statins, partial fatty acid oxidation inhibitors, and proton pump inhibitors. This medication profile reflects a holistic approach to cardiovascular health, addressing both hemodynamic and metabolic factors. Diagnostic tests for coronary artery disease included blood tests (additionally blood sugar and cholesterol level measurements), electrocardiograms (ECGs), and echocardiograms, and some patients had heart CT scans to produce detailed images of the heart and blood vessels. The research study spanned 4 weeks, with patient numbers and average ages across subgroups detailed in Table 1. Written informed consent was obtained from all participants before their involvement. Exclusion criteria included individuals under 18, pregnant women, those with ACS, autoimmune diseases, a history of pancreatic tumors or previous pancreatic disorders, severe anemia (hemoglobin < 7 g/L), hypokalemia (serum K^+^ < 3.5 mmol/L), acute renal failure, or active gastrointestinal bleeding, and non-consenting participants. The study adhered to the Declaration of Helsinki and was approved by the Ethics Committee of the University of Pécs (permission number: 3362/5636–PTE–2017/23).

### 2.2. Coronarography Procedure and Stent Implantation

Catheterization is used for the diagnosis and treatment of various heart disorders. An extensive range and variety of small instruments can be advanced through the catheter to perform specific diagnostic and therapeutic tasks (devices for measuring blood pressure in each heart chamber and also the major blood vessels connected to the heart, as well as tools for obtaining ultrasound images of the interior of blood vessels). The procedure enables thorough evaluation and accurate intervention during its course. Coronarography (coronary angiography) is a diagnostic procedure in which contrast dye is injected into the coronary arteries to enable fluoroscopic visualization. This allows for real-time X-ray imaging to identify any obstructions or blockages within the coronary arteries. The procedure is performed in a hospital setting and typically takes between 40 to 60 min to complete. A local anesthetic is administered to anesthetize the site of insertion. A catheter is then introduced into the heart via the radial, femoral, or brachial artery to facilitate access to the coronary circulation. Continuous X-ray images are taken, and any stenoses or occlusions are identified. If indicated, stents—small, permanent metal tubes—are inserted into narrowed arteries to restore adequate blood flow. The degree of stenosis varied among the patients, influencing the type and necessity of stents they received (in our case: 4 patients 50%, 3 patients 75%, and 1 patient subtotal). Indication for stent implantation was assessed visually; if the grade of stenosis was higher than 50%, a stent was implanted. In total, 5 patients had stenosis in the left anterior descending artery (LAD) and 3 had stenosis in the circumflex artery (CX), (5 were implanted with 2 stents for LAD and CX). This procedure provides valuable insights into coronary vessels and heart function as well. It is typically minimally discomforting, with most patients experiencing little to no severe pain. Serious complications, including cardiogenic shock, seizures, renal impairment, and cardiac arrest, are exceedingly rare. Side effects of radiopaque contrast agents can include allergic reactions (from mild skin rashes to rare life-threatening anaphylaxis) and nephrotoxicity. Contrast-induced nephropathy typically resolves spontaneously. Hospitalization typically lasts one to two days, during which patients receive comprehensive information and provide informed consent prior to admission.

### 2.3. Medication and Contrast Material

Before the intervention, patients were briefed on the procedure, risks, and complications, and gave informed consent. Vital signs, including blood pressure, heart rate, and oxygen saturation, were continuously monitored. As a venous access, a tiny intravenous canula (Vasofix, B. Braun Medical, Melsungen, Germany) was inserted for the medication, dye (iodixanol), and fluid. A sheath (6F) was inserted into the left wrist radial artery for coronary catheterization. The entry site was cleaned with Betadine (Betadine, Egis Gyógyszergyár Kft., Budapest, Hungary) and numbed with local anaesthetic (Lidocaine 1%, Egis Gyógyszergyár Kft., Budapest, Hungary) to reduce pain. A long, flexible coronary catheter (JL3.5-4 OSH, EBU 3.5 SH, Medtronic Hungary Kft, Budapest, Hungary) was then inserted through the sheath into the blood vessel and carefully guided via real-time X-ray to reach the coronary arteries. The iodine-based contrast dye (Visipaque, GE Healthcare, Chicago, IL, USA) injected through the catheter enabled visualization of the blood vessels, specifically the coronary arteries, on X-ray images. The procedure enabled the medical team to evaluate blood flow and identify any obstructions or irregularities within the coronary arteries. Continuous imaging was performed as a contrast agent was administered, enhancing the visualization of the coronary arteries and allowing for a precise assessment of their dimensions, morphology, and patency. Pressure measurements within the heart chambers and arteries may also be performed during the procedure to evaluate cardiac function. In cases where intervention was required, stents were inserted (Onyx, Medtronic Hungary Kft, Budapest, Hungary and Synergy, Boston Scientific, Marlborough, MA, USA) into the coronary arteries. The number of stents inserted depended on the extent of narrowing and the characteristics of abnormalities detected. After the procedure, the catheter was removed, and the entry site was sealed with either a closure device or manual compression using Cosmopor E (Hartmann-Rico Hungary Kft., Budapest, Hungary) to prevent bleeding. Patients were monitored for complications like bleeding, hematoma, or allergic reactions. If a stent was placed, patients could often be discharged the day after coronary angiography. Two medications were given to prevent stent clotting: one with acetylsalicylic acid (Astrix, Teva Pharmaceutical Industries Ltd., Budapest, Hungary) and the other with “Thienopyridine” (Plavix, Sanofi Winthrop Industries, Paris, France).

### 2.4. Blood Sampling

Blood samples were collected from each patient at four specific time points during the procedure to measure SST-like immunoreactivity (SST-LI) in the plasma. The sampling schedule was as follows: the first sample (sample A) was taken at the very beginning before the intervention commenced; the second sample (sample B) was collected immediately after the completion of coronary angiography; the third sample (sample C) was drawn two hours from the start of the procedure; and the fourth sample (sample D) was obtained the following morning at 6:00 a.m. This meticulous timing allowed for a comprehensive assessment of SST-LI levels throughout the course of the intervention and the immediate post-procedural period. Immediately after collection, 3-milliliter blood samples were placed into ice-cold vacutainers containing EDTA (18 mg REF 367525 and 143 I.U. REF 367674) to prevent clotting. Additionally, to prevent enzymatic degradation and ensure the accuracy of somatostatin measurement, 200 µL of the peptidase inhibitor aprotinin (Trasylol, Bayer HealthCare, Leverkusen, Germany) was promptly added to each blood sample designated for SST-like immunoreactivity (SST-LI) analysis. This step was crucial in preserving the integrity of the peptide hormones in the plasma, allowing for reliable and consistent measurement of SST-LI levels across all collected samples. After centrifugation at 1000 rpm for 5 min followed by 4000 rpm for 10 min, the plasma samples were promptly frozen at −70 °C to maintain stability until further analysis. This rigorous storage protocol ensured the preservation of sample integrity and consistency in subsequent assessments. All samples were analyzed under standardized conditions at the conclusion of the study, adhering to strict protocols to minimize variability and ensure accurate measurement of SST-LI. Previous studies have shown that accurate determination of SST-LI using the ELISA method typically necessitates the use of 3 mL of whole blood, from which 100 µL of plasma is subsequently obtained. The procedures were conducted without sedation, and patients adhered to hospital protocols by fasting from food and drink after midnight preceding the intervention. This fasting regimen ensured standardized conditions for the procedure, reducing and minimizing potential variables related to sedative use or dietary intake.

### 2.5. Determining Plasma SST-LI

Enzyme-Linked Immunosorbent Assay (ELISA) is a widely used laboratory technique for sensitive and specific detection of molecules. The CEA 592 Somatostatin ELISA kit (Cloud-Clone Corp., Katy, TX, USA) was utilized for analysis. This assay demonstrates high sensitivity and excellent specificity in detecting SST-LI. Before measurement started, the components and samples were kept at room temperature, and all reagents, samples, and standards were prepared. Fifty microliters of standard and sample were added to each well, followed by the immediate addition and agitation of 50 µL of prepared Detection Reagent A. The mixture was then incubated for 1 h at 37 °C. After aspiration, the wells were washed three times, and 100 µL of prepared Detection Reagent B was added, followed by another incubation period of 30 min at 37 °C. Subsequently, the wells were aspirated and washed five times. Ninety microliters of substrate solution was then added and incubated for 10–20 min at 37 °C. Finally, 50 µL of stop solution was added, and the reading was taken immediately at 450 nm. Using standards of known concentrations, the concentration of the substance (SST) in the solution was calculated from the light absorbance (concentration). By comparing the absorbance values of the samples to a standard curve generated from the known concentrations of the standards, the concentrations of SST-LI in the samples were determined. Controls are included in each ELISA plate to ensure the accuracy and reproducibility of the results. This ELISA method provides quantitative measurements of SST-LI levels in plasma samples, offering valuable insights into its role in various physiological and pathological processes related to coronary artery disease and inflammation.

### 2.6. Statistical Analysis

SST-LI values expressed in pg/mL were log-transformed for statistical analysis. Statistics were performed using R language (version 4.2.2) and RStudio (v2023.06.2). Effect sizes were calculated and interpreted using the effect size (version 0.8.6) R package [29,30,31]. *p*-values < 0.05 were considered significant. Linear mixed model analysis was run using the lme4 package (Version: 1.1-35.5) [32]. We included SST-LI as the dependent variable and added fixed effects of sampling time points (A, B, C, D) as well as coronarography only and stent implantation. We included patients as a random effect. Significance was calculated by applying Satterthwaite’s method to estimate degrees of freedom and generate *p*-values for mixed models. The model specification was as follows: SST-LI ~ Time * Intervention + (1|Patient). For complete observations of patients with SST-LI measured at all four time points (n = 7), repeated measures ANOVA was calculated. Post hoc power value analysis was performed for all statistical tests to justify the sample sizes, and the effect size values were calculated to determine differences between the groups incorporating the sample size as well. Effect size indicates how meaningful the relationship between variables or the difference between groups is, showing the practical significance of a research outcome. A large effect size (Hedges’ g > 0.8; Cohen’s f > 0.4) means that the research finding has practical significance, while a small effect size indicates limited practical applications [33,34]. Dunnett’s many-to-one comparison test was applied using the PMCMRplus package (v1.9.10). Spearman’s rank correlation was calculated using the psych package (v2.3.9), effect size was calculated using effect size v0.8.9, and post hoc power was calculated using pwr 1.3-0 and WebPower v0.9.4.

## 3. Results

### 3.1. Plasma SST-LI Significantly Decreases with Aging in the Healthy Population

**Figure 1:**
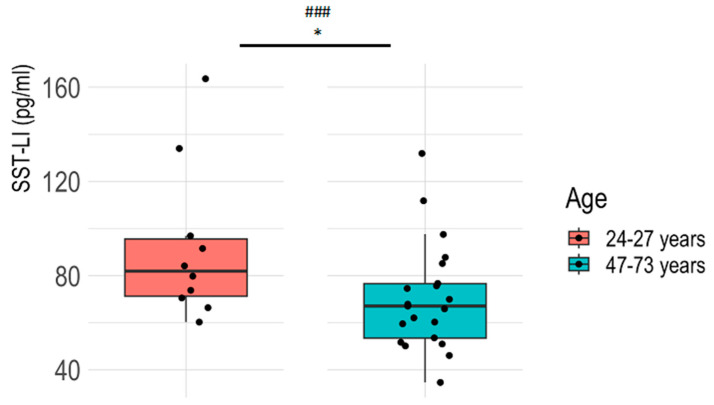
Plasma SST-LI in healthy volunteers in two age groups. In the boxplots, summary statistics are visualized for the two groups, 24–27 years (n = 10) and 47–73 years (n = 21): the median, two hinges (lower: first quartile or 25th percentile; upper: third quartile or 75th percentile), and two whiskers. The upper whisker extends from the hinge to the largest value no further than 1.5×. Inter-quartile range (IQR: the distance between the first and third quartiles). The lower whisker extends from the hinge to the smallest value at most 1.5 × IQR of the hinge. ### represents a large effect size, * *p* < 0.05 (Welch’s *t*-test for unpaired comparison). The results of the statistical evaluation are provided in Table S1.

Plasma SST-LI was significantly higher in young, healthy controls (24–27 years) compared to older ones (47–73 years), determined via the Welch two-sample *t*-test. The difference proved to have a large effect size [29]. There was a strong negative correlation between SST-LI concentrations and age (Figure 1).

### 3.2. Plasma SST-LI Is Significantly Lower in IHD Patients Compared to Age-Matched Healthy Controls

**Figure 2:**
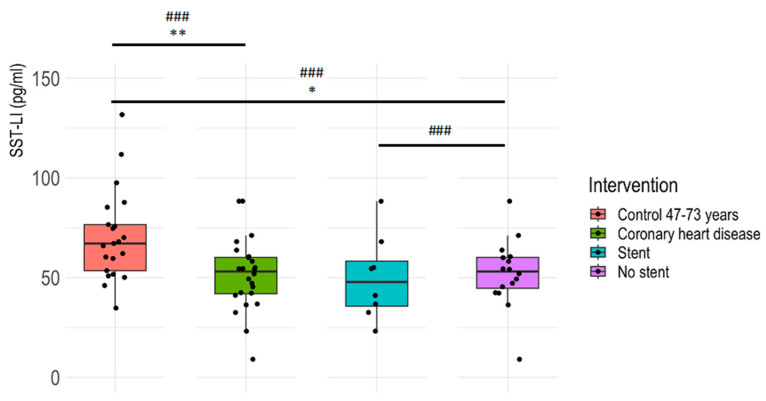
Plasma SST-LI levels (pg/mL) in age-matched controls and in coronarography patients without stents and with stents before intervention. Age-matched healthy controls (47–73 years, n = 21), no-stent patient group (n = 16), and stent patient group (n = 8). Effect size: ### large; *p*-value: ** *p* < 0.01, * *p* < 0.05. In the boxplots, five summary statistics are visualized: the median, two hinges (lower: first quartile or 25th percentile; upper: third quartile or 75th percentile), and two whiskers. The upper whisker extends from the hinge to the largest value no further than 1.5 * IQR from the hinge (where the IQR is the inter-quartile range or the distance between the first and third quartiles). The lower whisker extends from the hinge to the smallest value, at most 1.5 * IQR of the hinge.

Plasma SST-LI was significantly lower in the IHD patients before coronarography compared to their age-matched healthy controls. In patients without stents, there was only a decreasing tendency, but a large effect size was observed when compared to those with stents. ANOVA *p*-value: 0.02115. Dunnett’s post hoc test: no-stent group vs. age-matched controls *p*-value = 0.0308; stent group vs. age-matched controls *p*-value = 0.0569 (Figure 2). For detailed statistical results, please see Supplementary Table S2.

### 3.3. Plasma SST-LI Significantly Increases in IHD Patients 24 Hours after Stent Insertion

**Figure 3:**
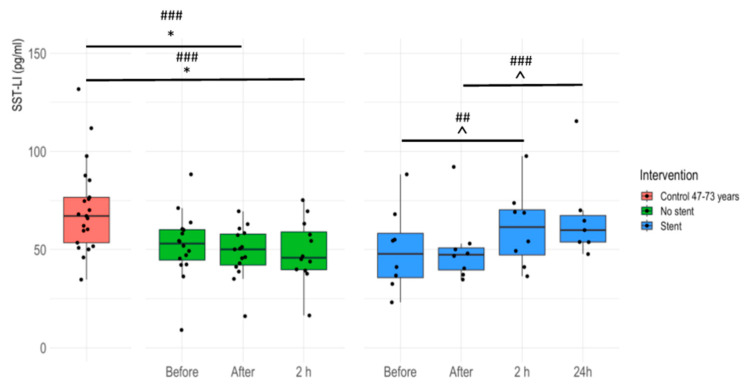
Plasma SST-LI in patients having coronarography with and without stent implantation before, right after, 2 h, and 24 h following the intervention compared to the age-matched healthy controls. Boxplots demonstrate the medians, two hinges (lower: first quartile or 25th percentile; upper: third quartile or 75th percentile), and two whiskers with the greatest value no further than 1.5 * inter-quartile range (IQR) from the hinge (where the IQR is the distance between the first and third quartiles and the lower whisker extends from the hinge to the smallest value at most 1.5 * IQR of the hinge. Effect size: ### large; ## moderate. Dunnett’s *p*-value: * *p* < 0.05. Pairwise *t*-test for stent patients *p*-value: ∧ *p* < 0.05.

Plasma SST-LI values significantly decreased in patients without stent insertion right after (‘After’) and 2 h after the intervention compared to age-matched controls. In stent-receiving patients, SST-LI increased significantly at 24 h compared to time point ‘After’, as well as at 2 h compared to ‘Before’ (Figure 3).

For detailed results, please see Supplementary Table S3.

There was no significant difference in SST-LI levels between patients with differing stent numbers or stent positions. No significant correlation was found between stent size and SST-LI values. In the stent group, there was no statistical correlation between either SST-LI and ischemic time or SST-LI concentrations and plasma laboratory parameters (Na^+^, K^+^, CN, creatinine, GFR, troponin, and Mg^++^) 24 h after the intervention (Pearson correlation on log-transformed SST-LI values and Spearman’s rank correlation with Benjamini–Hochberg correction for multiple comparisons). The highest absolute correlation coefficient (−0.70) was found between SST-LI and Na^+^ levels (Spearman’s correlation, *p*-value: 0.07). Independently of SST-LI, a significant negative correlation was found between troponin and magnesium levels (Pearson’s correlation, r: −0.83, *p*-value: 0.02).

## 4. Discussion and Conclusions

Herein, we provide the first human data that plasma SST-LI significantly decreases with age; it is lower in IHD patients and increases after coronary stent implantation.

Our results are supported by rodent immunohistochemistry data showing that SST levels decrease with age in the median eminence of male rats at 3, 12, 20, and 30 months [35]. It has been demonstrated that age-related SST decrease is a consequence of lower gene expression within specific hypothalamic nuclei [36]. Human studies have revealed that impaired neuropeptide homeostasis may play a significant role in age-related disorders. For instance, the age-related dysregulation of endogenous PACAP-dependent pathways [37] could contribute to cerebrovascular impairments in aging.

Previous research indicates that neuronal signaling profoundly impacts tissue regeneration following traumatic injuries [38]; the neuroprotective benefits of SST in diabetic retinas are primarily due to its anti-inflammatory properties [39].

SST-LI was detected in the human heart (interventricular septum, left ventricle), which was significantly lower in ischemic cardiomyopathy samples than in samples from healthy controls. SST and SST receptors were not identifiable at the mRNA level through sequencing in the left ventricles of healthy humans; however, SST is present in heart tissue at the peptide level. Therefore, the origin of SST is likely to be sensory neural, where cell bodies with mRNA are located in the dorsal root ganglia. SST expression was decreased at the peptide level in the interventricular septum samples of patients with ischemic cardiomyopathy [15]. 

The Transient Receptor Potential Vanilloid 1 (TRPV1) capsaicin receptors located on the peptidergic sensory nerves that supply the heart [40] can be activated and/or sensitized by substances associated with tissue damage and inflammation, such as protons and prostaglandins [26]. This stimulation might result in the release of sensory neuropeptides, including SST, which enter the systemic circulation during reperfusion processes. Coronary stent implantation as a therapeutic intervention in patients with IHD induces reperfusion and potentially causes subsequent myocardial microinflammation. This cascade of events might result in SST outflow from the peptidergic capsaicin-sensitive sensory nerves of the heart and/or recruited and accumulated inflammatory cells in response to tissue injury and inflammatory mediators. SST might exert inhibitory effects against oxidative stress and inflammation as a protective mechanism. Although our descriptive results do not directly explain this concept, a wealth of previous animal experimental data in a broad range of inflammation and pain models might support this theory [27,41,42,43]. In a previous study, the highest SST concentrations were found in the atrioventricular node and right atrium with a similar distribution in humans and guinea pig models [44] via radioimmunoassay. During and after stent implantation, the significantly improved coronary blood flow can be attributed to the release of detectable SST increase in human plasma. This inference is supported by the fact that blood samples were obtained after a 12 h period of fasting, making gastrointestinal release unlikely [45].

Recent studies have demonstrated that elevated levels of N-Terminal prosomatostatin (NT-proSST) are associated with a higher incidence of cardiovascular diseases and increased all-cause mortality [46], as well as with the onset of peripheral arterial disease [47]. Neuropeptides have been suggested to be potential novel prognostic biomarkers in addition to the traditional indicators such as troponins and natriuretic peptides in cases of cardiac injury [48]. Coronary microvascular dysfunction, a commonly observed phenomenon in cardiology, arises from endothelial damage with systemic inflammation playing a significant role linked to various comorbidities [49]. The inflammatory process in the atherosclerotic artery may lead to increased blood levels of inflammatory cytokines and other acute-phase molecules [50]. The level of active inflammation intensifies in activated plaques found in patients with acute coronary syndromes. Additionally, these lesions may release inflammatory mediators into the systemic circulation [51]. Monocytes and macrophages are pivotal in triggering vascular inflammation, driving atherosclerosis by promoting necrotic core formation, thinning fibrous caps, and increasing plaque vulnerability [52,53]. The interplay between the stage of plaque development, macrophage density and activity, and the expression of specific SSTR subtypes has been demonstrated [54]. The involvement of SST in inflammation is intricate, encompassing various signaling molecules and pathways within both neuronal and immune cells. Unraveling the specific role and effects of SST within this complex network is challenging. Inflammatory and immune cells like macrophages and lymphocytes release an array of signaling molecules including cytokines, chemokines, and additional neuropeptides upon activation. SST reduces the release of inflammatory mediators, such as cytokines and histamine from immune cells [55]. Understanding the multifaceted interactions between SST and myocardial ischemia and reperfusion after coronary stent implantation with the various components of the immune and nervous systems provides valuable insights into the potential therapeutic applications of SST or more likely its stable, selective analogs. During reperfusion, where blood flow is restored after a period of ischemia, SST may influence the inflammatory response and oxidative stress, potentially impacting tissue recovery and outcomes following myocardial damage and infarction. Understanding the role of SST in IHD and reperfusion injury is crucial for exploring potential therapeutic strategies aimed at improving outcomes in cardiovascular patients. 

Our research has some limitations, such as the relatively modest sample size and lack of details regarding the pre-intervention medication, which is one of the limitations of the study, but the data do not vary much and provide statistically significant differences and/or effect size values between the groups. Therefore, pharmacotherapy is not likely to substantially influence the baseline SST-LI data. The study included patients diagnosed with conditions like hypertension, previous MI, elevated lipid and cholesterol levels, and type II diabetes. Despite our rigorous selection criteria aimed at ensuring accurate patient selection and homogeneity within the study group, the presence of these diverse comorbidities could potentially influence our results. In the future, we plan to expand the patient cohort and conduct additional investigations, including subgroup analyses based on pharmacotherapy. Additional data could provide insights into the complex role of SST in coronary artery disease and inflammation, potentially paving the way for its clinical implementation as a significant factor in IHD management. 

Through our study, we have established a link between SST-LI levels and ischemic heart disease, as well as the alteration of SST-LI in the plasma of patients with coronary stent implantation. These results contribute to a more comprehensive understanding of the neuropeptidergic sensory regulation of the myocardium and highlight its potential role under ischemia–reperfusion conditions. The findings of the present study may have potential clinical relevance as a valuable marker for coronary stent implantation and an opportunity for novel therapeutic treatment strategies via SST signaling.

## Figures and Tables

**Table 1:** Number and mean age ± SD of patients and controls in the different subgroups going through the intervention procedure.

	Healthy Controls	Healthy Control Group 1.	Healthy Control Group 2. (Age Matched)	Patients for Coronarography	Coronarography without Stent Implantation	Coronarography with Stent Implantation
Male	11	5	6	12	8	4
Female	20	5	15	12	8	4
Total	31	10	21	24	16	8
Mean age ± SD	43.74 ± 15.32	23.3 ± 0.82	53.47 ± 6.6	68 ± 9.29	66.06 ± 8.51	72.125 ± 9.74

## Data Availability

All data created or analyzed during this study are available from the corresponding author upon request.

## Supplementary Materials

The following supporting information can be downloaded at: https://www.mdpi.com/article/10.3390/jcm13164727/s1. Table S1: Plasma SST-LI in healthy volunteers in two age groups; Table S2: Plasma SST-LI levels (pg/mL) in age-matched controls and in coronarography patients without stents and with stents before intervention; Table S3: Plasma SST-LI in patients having coronarography with and without stent implantation before, right after, 2 h, and 24 h following the intervention compared to the age-matched healthy controls.

## Glossary

SST = somatostatin; SST-LI = somatostatin-like immunoreactivity; NT-proSST = N-terminal prosomatostatin; SSTR = somatostatin receptors; ELISA = Enzyme-Linked Immunosorbent Assay; CHD = coronary heart disease; IHD = ischemic heart disease; CVD = cardiovascular disease; MI = myocardial infarction; LAD = left anterior descending artery; CX = circumflex artery; PAD = peripheral arterial disease; TRPV1 = Transient Receptor Potential Vanilloid 1; TRPA1 = transient receptor potential ankyrin 1; mRNA = messenger ribonucleic acid; ACE = angiotensin converting enzyme.
