# Supplementary material for: Plasma Somatostatin Levels Are Lower in Patients with Coronary Stenosis and Significantly Increase after Stent Implantation
Source: J Clin Med. 2024 Aug 12;13(16):4727. doi: 10.3390/jcm13164727 (PMC11355572; doi:10.3390/jcm13164727)
Supplement: Supplementary file 1 [file jcm-13-04727-s001.zip › Tables S1-S3.pdf]

Table S1. Plasma SST-LI in healthy volunteers in two age groups.

	24-27 years	47-73 years
Mean SOM-LI (pg/ml)	92.04	70.53
95% CI for difference in means (log)	0.0120 – 0.5175	
Effect size - Hedges' g	0.80, large	
95% confidence interval for effect size	0.03 – 1.55	
Welch two sample t-test p-value	0.0411	
Welch two sample t-test post-hoc power	0.52	
Spearman correlation (age, SST-LI) r	-0.55	
Spearman correlation (age, SST-LI) p-value	0.0013	
Spearman correlation (age, SST-LI) post-hoc power	0.91	

CI: confidence interval

Table S2. Plasma SST-LI levels (pg/mL) in age-matched controls and in coronarography patients without stents and with stents before intervention.

	Control 47-73 years	No stent	Stent
Mean (pg/ml)	70.53	52.14	49.92
Coronary disease (stent and no stent) mean (pg/ml)		51.40	
Effect size - Hedges' g		0.92 vs. control	
95% confidence interval for effect size		0.31 – 1.52	
Effect size interpretation - [30]		large	
Welch Two Sample t-test p-value		0.0045	
Welch Two Sample t-test power		0.54	
95% CI for difference in means (log)		0.12 – 0.59	
Effect size - Hedges' g		0.88 vs. control	0.89 vs. no stent
95% confidence interval for effect size		0.20 - 1.54	0.06 –1.71
Effect size interpretation - [30]		large	large
ANOVA (control, no stent, stent) p-value	0.0212		
Adjusted R-squared	0.1281		
F-statistic	4.233		
ANOVA effect size – Cohen's f	0.45		
95% confidence interval for ANOVA effect size	0.016 – 1.000		
ANOVA effect size interpretation – Cohen	large		
ANOVA (control, no stent, stent) power	0.47		
Dunnett's-test for multiple pairwise comparisons with control group - p-value		0.0308	0.0569

Table S3. Plasma SST-LI in patients having coronarography with and without stent implantation before, right after, 2 h, and 24 h following the intervention compared to the age-matched healthy controls.

	Control 47-73 years	No stent - Before	No stent - After	No stent - 2h	No stent - 24h	Stent - Before	Stent - After	Stent - 2h	Stent - 24h
Mean (pg/ml)	70,53	52,14	48,45	49,07	(44,65)	49,92	50,30	61,26	66,47
Linear mixed model fixed effect p-value	After: 0.6574; 2h: 0.0157; 24h: 0.00546; No stent: 0.00671; Stent: 0.01456; No stent - After: 0.5506; No stent - 2h: 0.04712; No stent: 0.05774								
Dunnett's-test for multiple pairwise comparisons with control group - p-value	-	0.05005	0.02507	0.04116	-	0.10210	0.1881	1.0000	1.0000
Repeated measures ANOVA for complete observations (stent patients, n=7) p-value; post-hoc power						p: 0.0270; power: 0.05			
Pairwise t-tests for complete observations (stent patients, n=7) p-value with Benjamini-Hochberg correction; post-hoc power						Stent before vs. Stent 24h: p: 0.0490; power: 0.26	Stent after vs. Stent 24h: p: 0.0290; power: 0.41		
Effect size - Hedges' g		No-stent before vs. control: -0.88	No-stent after vs. control: -1.11	No-stent 2h vs. control: -1.01	-	Stent 24h vs. Stent before: 0.59	Stent 24h vs. Stent after: 0.68		
95% confidence interval for effect size		0.20-1.54	0.40-1.80	0.27-1.74	-	0.43-1.59	0.37-1.66		
Interpretation of effect size; [30]		large	large	large	-	moderate	large		
